# Interposition arthroplasty for post-traumatic osteoarthritis of the elbow: a systematic review

**DOI:** 10.1007/s00264-022-05562-3

**Published:** 2022-08-30

**Authors:** Fabian Lanzerath, Michael Hackl, Christoph-Johannes Pucher, Tim Leschinger, Stephan Uschok, Lars P. Müller, Kilian Wegmann

**Affiliations:** 1 Department of Orthopedic and Trauma Surgery, University Hospital Cologne, Kerpener Street 62, 50937 Cologne, Germany; 2 OCM (Orthopädische Chirurgie München) Clinic, Munich, Germany

**Keywords:** Interposition arthroplasty, Post-traumatic, Osteoarthritis, Elbow, Systematic review

## Abstract

**Purpose:**

Interposition arthroplasty for the post-traumatic osteoarthritic elbow is a salvage procedure used in young and active patients and remains a rare and unexplored therapeutic option.

**Methods:**

We systematically reviewed the available literature searching electronic databases, MEDLINE using the PubMed interface and EMBASE. The primary objective was to synthesize functional outcomes and to investigate revision frequencies, but also complication and subsequent surgery rates among patients with surviving grafts. The preferred reporting guidelines for systematic reviews and meta-analyses guidelines were applied.

**Results:**

Five studies were left for inclusion, all retrospective in design, comprising 67 patients. The mean age was 40 years, the mean follow-up period was 61 months, and 68.2% of the patients treated were male. Eleven patients (16.4%) were treated with fascia lata autografts, and 56 patients (83.6%) were treated with Achilles tendon allografts. The graft survived in 53 patients (79.1%); the post-operative Mayo Elbow Performance Score averaged 69 points. Fourteen patients (20.9%) required revision surgery. In the setting of graft survival, 39.1% of patients had complications not requiring further surgical treatment and 5.7% of patients with surviving grafts needed subsequent operative treatment within the follow-up period.

**Conclusion:**

Given graft survival, this systematic review demonstrated satisfactory functional outcomes following interposition arthroplasty of the post-traumatic osteoarthritic elbow, however, associated with a cumulative complication and subsequent operative treatment rate of 44.8%. In addition, a revision rate of 20.9% needs to be expected. Varus-valgus instability in the pre-operative clinical assessment seems to be associated with unsatisfactory post-operative elbow function. The superiority of either of the two main reported graft methods (fascia lata autograft and Achilles tendon allograft) remains pending, and the role of an external fixator in preventing post-operative instability remains unresolved.

## Introduction

Although performed for many years, interposition arthroplasty (IPA) still remains a rare therapeutic option. However, in young, high-demanding patients with sufficient bone stock, IPA seems to be a viable treatment alternative to total elbow arthroplasty (TEA), after non-operative treatment failed and arthrodesis is ruled out due to the functional limitations. Studies identified age < 65 years and trauma history as risk factors for prosthesis loosening after TEA [1]. Post-traumatic osteoarthritis appears to be the leading indication for IPA at present [2]. Post-traumatic elbow osteoarthritis in the young and active patient poses a challenge to the surgeon, not only in terms of the surgical procedure itself but especially with regard to the optimal therapeutic decision [2]. Accordingly, IPA can be thought of as a middle course that preserves the possibility of a further escalation stage in the sense of TEA, while maintaining elbow mobility [3]. Relative contraindications for IPA include gross instability or deformity, infection, skeletal immaturity, and insufficient flexor muscles [4]. Thus, IPA intends to preserve the elbows mobility and to reduce pain, while the strict weightlifting restrictions of TEA do not apply, and revision options are kept available.

Given the limited availability of studies, we decided to conduct a systematic review to obtain a synthesis of functional outcomes and potential complications.

**Fig. 1:**
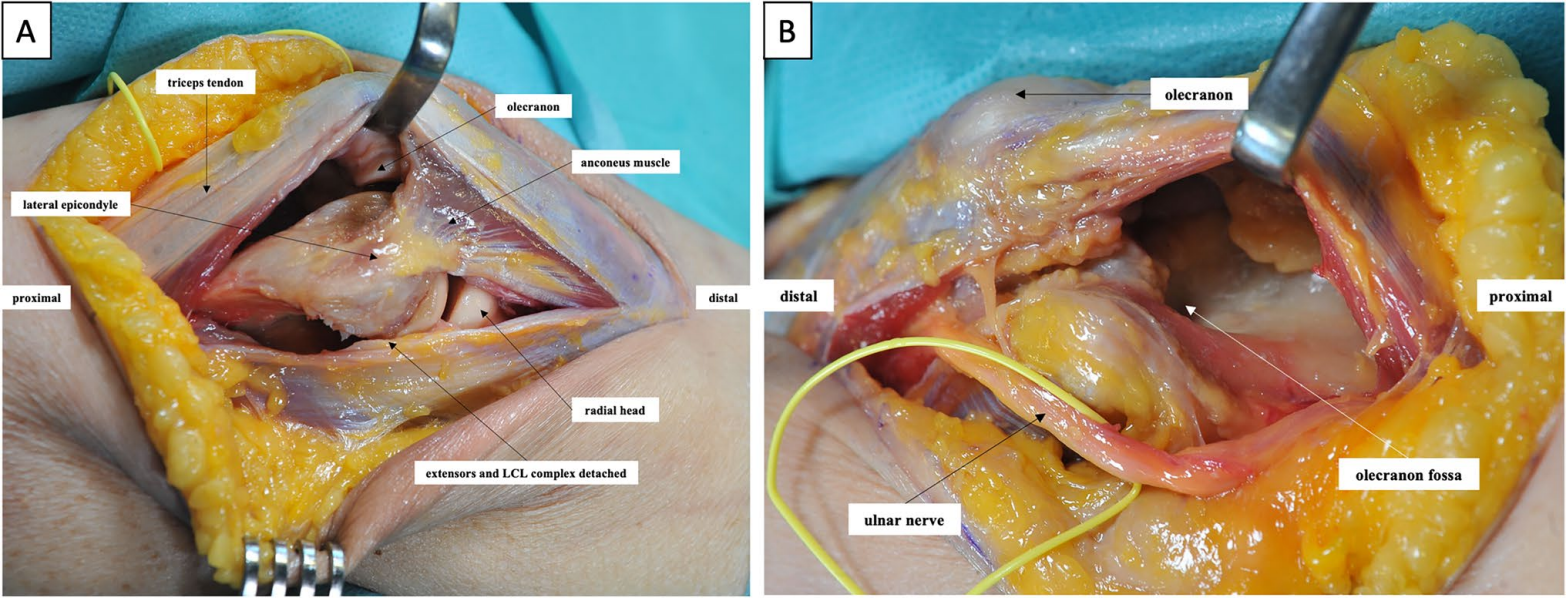
Approach and preparation of the joint for grafting. **A** After sketching the bony landmarks, the skin is incised posteriorly longitudinally with lateral circumcision of the olecranon. Laterally, the Kocher interval between the anconeus and flexor carpi ulnaris muscle is established. The capsuloligamentous attachments and extensor attachments are detached humerally. **B** Medially, the ulnar nerve is exposed, neurolyzed, and secured. Bilateral arthrolysis follows. The medial collateral ligament (MCL) should be preserved

**Fig. 2:**
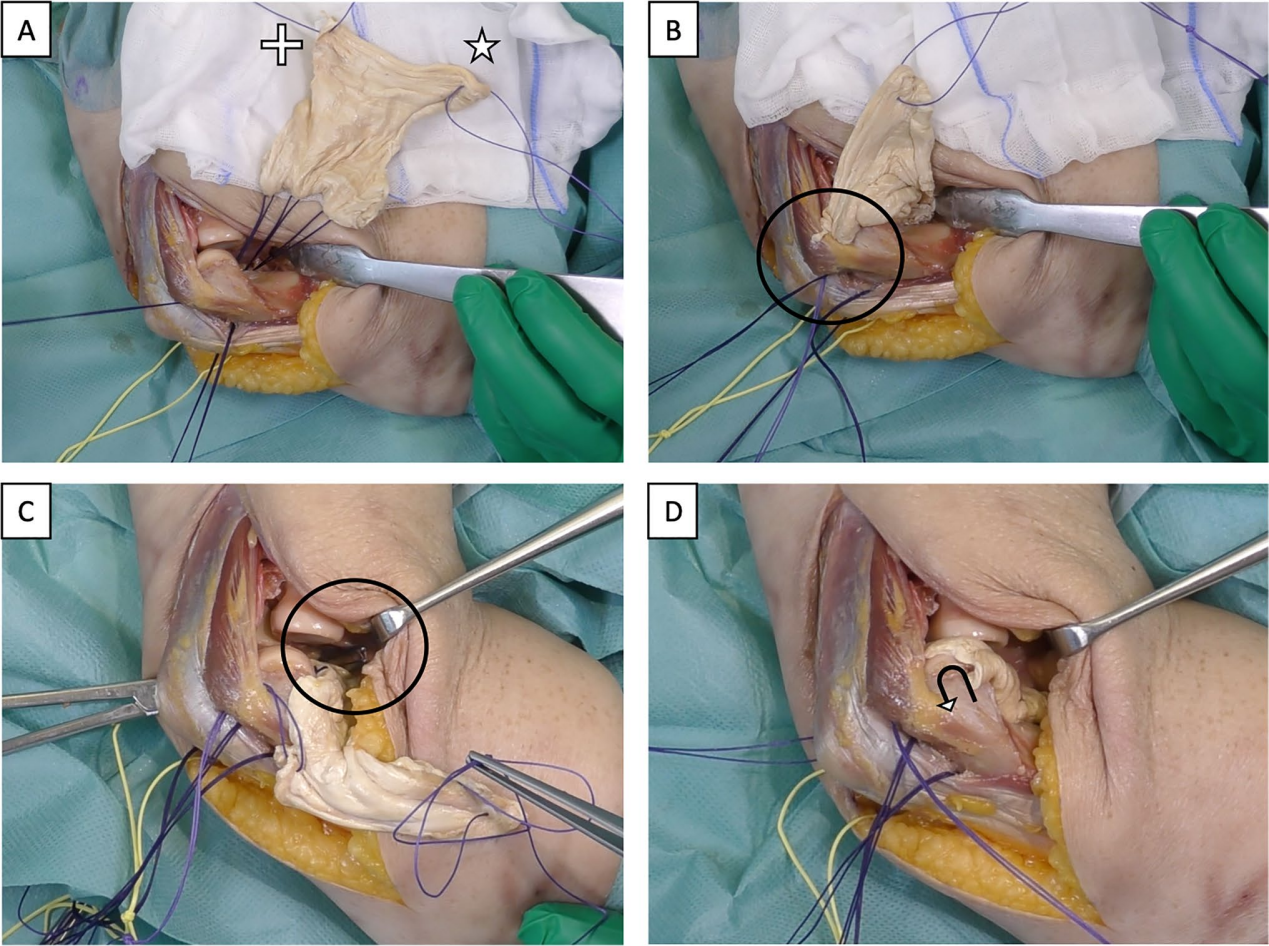
Placement of the graft. **A** Three transosseous drill holes are set in a dorsoventral direction in the distal humerus: one in the area of the lateral epicondyle and one each in the area of the lateral and medial olecranon fossa. A non-absorbable suture is inserted through each of the drill holes, again in a dorsoventral direction. The ventral end of each suture is looped through the graft, followed by stitching it back through the drill holes, now in a dorsoventral direction. To ensure that the graft is properly positioned later, it is looped with two pull-through sutures at its free corners. The lateral pull-through suture is marked with a plus, the medial suture with a star. **B** The lateral pull-through suture is passed dorsally under the anconeus muscle (circle). **C** The medial pull-through suture is guided dorsomedially by means of an Overholt (circle) inserted from the ulnar side. **D** By pulling on the two pull-through sutures, the graft slides from ventral over the articular surfaces to the dorsal aspect of the distal humerus, illustrated by the curved arrow. The correct position of the graft is to be verified

**Fig. 3:**
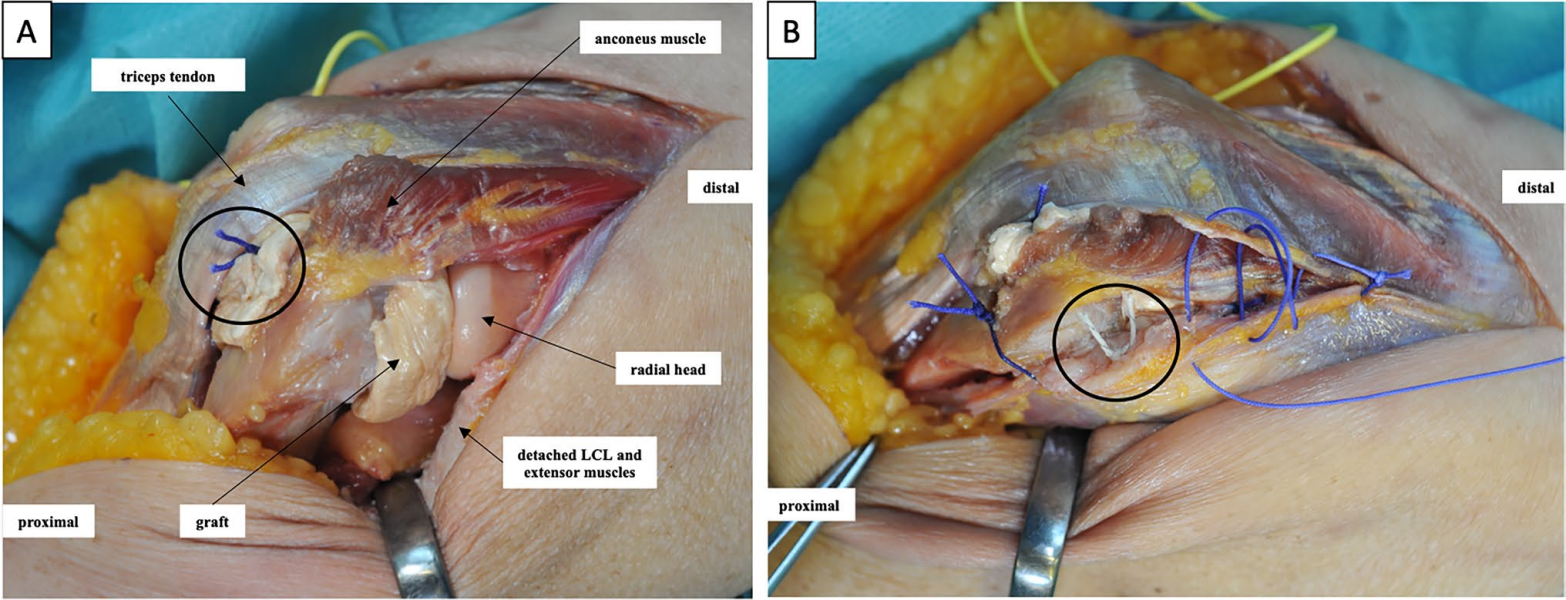
Grafts protection and reattachment of the lateral collateral ligament and the extensors. **A** With the three sutures pierced back through the drill holes to the dorsal side, the graft is now stitched once more from inside out. The sutures are then knotted onto the graft ensuring that it adapts to the dorsal aspect of the distal humerus (circle). **B** A suture anchor (circle) is inserted in the center of rotation to reattach the capsuloligamentous attachments and the extensor attachment, which have been detached humerally. The position of the interposition graft is checked again. Closure of the fascia, subcutaneous, and skin suture

At this point, the principles of the procedure are to be briefly explained in the authors’ own approach (Figs. 1, 2, and 3).

## Methods

The Preferred Reporting Items for Systematic Reviews and Meta-Analyses (PRISMA) guidelines were applied [5].

### Inclusion criteria

The following criteria for inclusion were defined preliminarily: (1) patients suffering from post-traumatic osteoarthritis (2) treated with IPA and in whom either (3) autogenous fascia lata or (4) Achilles tendon allografts were used as graft material, (5) studies published in English or German language since (6) the start of literature in the concerning electronic databases, and (7) providing the Mayo Elbow Performance Score (MEPS).

### Exclusion criteria

Patients treated with IPA for (1) primary, (2) inflammatory, or (3) rheumatoid osteoarthritis and (4) revisions were excluded.

### Search strategy

MEDLINE using the PubMed interface and EMBASE was searched for clinical studies using the MeSH terms interposition arthroplasty, elbow, and post-traumatic. The search was completed on January 5, 2021.

### Study selection

**Fig. 4:**
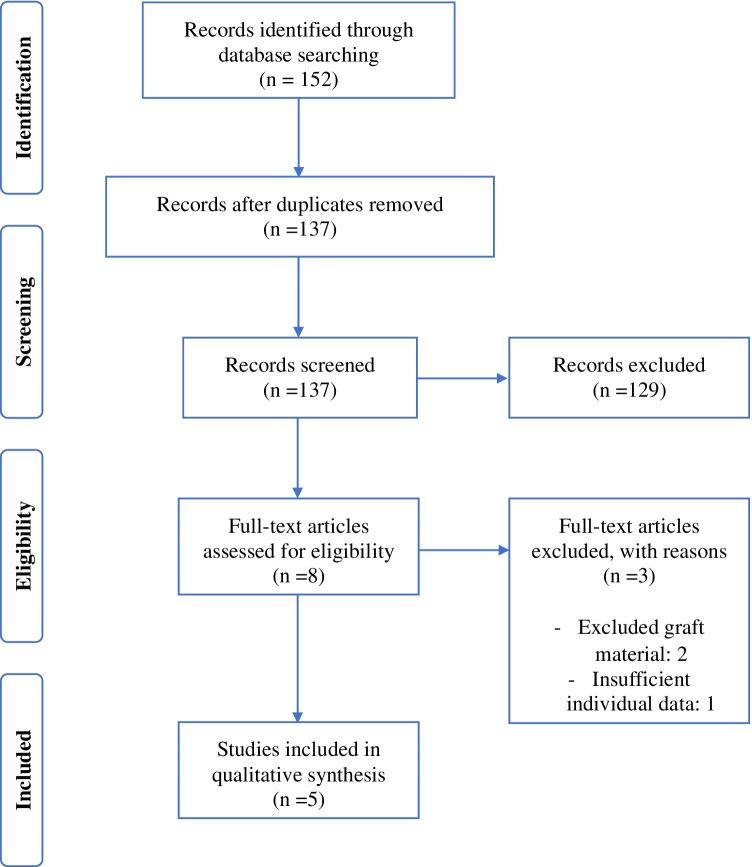
Study flow chart

The studies identified were independently scanned by 2 reviewers (F.L. and K.W.). At this stage, the titles and abstracts were assessed for eligibility. Full texts of the records, which outlived this process, were analyzed. Full texts’ reference lists were additionally analyzed and searched for further articles. This procedure is illustrated in the PRISMA-adapted flow diagram (Fig. 4). Disagreement was resolved by consensus decision including a third reviewer (L.P.M.).

By this means, a total of 5 studies were suitable for inclusion.

### Data extraction

The data of these five studies were extracted into prefabricated tables. The level of evidence was graded for each article included. The primary objective was to synthesize functional outcomes and to investigate revision frequencies, but also complication and subsequent surgery rates among patients with surviving grafts.

### Methodological quality

The methodological quality of each study included was assessed by assigning levels of evidence as previously defined by the Centre for Evidence-Based Medicine (http://www.cebm.net). Levels of evidence were assigned by two authors (F.L. and K.W.). If there was any disagreement, a third author was consulted (L.P.M.). Additionally, the methodological index for non-randomized studies (minors) items were applied [6]. This is a validated instrument that attributes a maximum score of 16 points to non-cooperative studies based on 8 items.

## Results

### Study selection

The initial search covered 152 publications. Removal of duplicates and exclusion of abstracts not fitting the inclusion criteria left 8 full texts for eligibility assessment. Three were excluded. Reasons are outlined in the PRISMA-adapted flow diagram (Fig. 4). Five publications were suitable for inclusion, comprising 67 patients [7–11]. Within the five studies included, an additional 26 patients were excluded based on the predefined inclusion and exclusion criteria. The level of evidence of all studies included was level IV. The studies of Cheng et al., Nolla et al., and Laubscher et al. all scored 9 points according to the minors criteria, while the studies of Larson et al. and Erşen et al. scored 10 points each.

### Study characteristics

The characteristics of the studies included are shown in Table 1. Among the 67 patients included, the mean age was 40 years, the mean follow-up was 61 months, and 68.2% of the patients treated were male. Eleven patients (16.4%) were treated with fascia lata autografts, and 56 patients (83.6%) were treated with Achilles tendon allografts. In all patients, total joint resurfacing was performed.

**Table 1:** Summary of the studies included

Author	Level of evidence	Patients included (*n*)	Patients excluded (*n*)	Mean age, years (range)	Mean FU, months (range)	Gender, % male	Graft material
Cheng et al	IV	9	4	36 (26–50)	48 (10–121)	38.5%*	9 Fascia lata autografts
Larson et al	IV	34	11	41 (16–69)	72 (35–126)*	79.4%	34 Achilles tendon allografts
Nolla et al	IV	13	0	41 (19–68)	48 (12–132)	69.2%	11 Achilles tendon allografts
							2 Fascia lata autografts
Erşen et al	IV	3	2	32 (26–41)	106 (84–131)	40%*	3 Achilles tendon allografts
Laubscher et al	IV	8	9	45 (35–58)	35 (8–57)	63%	8 Achilles tendon allografts
		67	26	40	61	68.2%	11 Fascia lata autografts (16.4%)
							56 Achilles tendon allografts (83.6%)

^*^Value applies to the whole study population (patients included and excluded)FU, follow-up

### Functional outcomes

The functional outcomes always refer to those patients whose graft survived and are summarized in Table 2. Relative to the most recent follow-up of each of the respective studies, the graft survived in 53 patients (79.1%). The average pre-operative MEPS was available in 42 patients and amounted to 39 points. The average post-operative MEPS was available in 49 patients and amounted to 69 points, which corresponds to an average improvement of approximately 57%. Excellent or good results concerning the MEPS were rated as successful treatment. Individual data regarding this were available for 22 patients with surviving grafts post-operatively. Accordingly, 16 of the available 22 patients (73%) were treated successfully. In the study performed by Nolla et al., a post-operative MEPS was obtained in only seven patients, although the graft survived in 11 patients. In the remaining studies, the average post-operative MEPS was always obtained in patients with surviving graft, although individual data (excellent, good, fair, or poor) were not always available.

**Table 2:** Summary of the functional outcome scores

Author	Patients included (*n*)	Outcome good–excellent**	Mean preop. MEPS (range) (*n*)**	Mean postop. MEPS (range) (*n*)**	Mean preop. flex.-ext.** (*n*)	Mean postop. flex.-ext.** (*n*)	Mean preop. forearm rotation** (*n*)	Mean postop. forearm rotation** (*n*)
Cheng et al	9	5/6	37 (20–50) (6)	76 (20–100) (6)	63° (6)	92° (6)	NA	NA
Larson et al	34	NA	40 (5–60) (27)	62 (30–100) (27)	NA	NA	NA	NA
Nolla et al	13	6/7	NA	81 (60–100) (7)	37° (11)	110° (11)	78° (11)	148° (11)
Erşen et al	3	1/3	27 (20–35) (3)	68 (60–75) (3)	27° (3)	70° (3)	8° (3)	52° (3)
Laubscher et al	8	4/6	44 (30–60) (6)	77 (40–100) (6)	NA	NA	NA	NA
	67	16/22	39 (42)	69 (49)	43° (20)	99° (20)	63° (14)	127° (14)

^**^Value applies to patients with surviving grafts only

*MEPS*, Mayo Elbow Performance Score; *NA*, not available; *preop.*, preoperative; *postop.*, postoperative; *flex. ext.*, flexion–extension

### Complications

Fourteen patients (20.9%) required revision surgery (Table 3). This involved switching to TEA most frequently (8 TEA, 2 graft removal, 2 arthrodesis, 1 revision IPA, 1 NA). Among the patients with surviving grafts, nine (39.1%) had complications not requiring further operative treatment, the most common being pin related problems of the external fixator. This average only refers to those patients whose graft survived and only to those studies in which the listed complications could be attributed exclusively to patients with post-traumatic osteoarthritis (individual data or data of the post-traumatic osteoarthritis cohort of the respective study were thus given). Complications were considered to be any listed as a complication in the individual studies. Three patients (5.7%) with surviving grafts needed subsequent operative treatment within the follow-up period.

**Table 3:** Summary of the complications

Author	Patients included (*n*)	Revision surgery (*n*)	Complications (*n*)**	Subsequent operative treatment (*n*)**
Cheng et al	9	3 TEA	1 ulnar nerve paresthesia (1 pre-existing)	1 ulnar nerve paresthesia (subcutaneous transposition, resolved)
			1 fascia lata donor site discomfort	1 fascia lata donor site infection with muscle herniation (fascial defect repaired, resolved) and superficial radial nerve paresthesia (resolved)
			1 pin-site infection (pin removal, resolved)	
Larson et al	34	4 TEA	NA	0
		2 Graft removed		
		1 Arthrodesis		
Nolla et al	13	1 Arthrodesis	2 pin-site infection (antibiotics, resolved)	1 ulna fracture requiring ORIF
		1 NA		
Erşen et al	3	0	NA	0
Laubscher et al	8	1 TEA	1 deep sepsis	0
		1 Revision IPA	1 wound sepsis, fracture through pinsite	
			1 ulnar nerve paraesthesia	
			1 instability	
	67	14 (20.9%)	9 (39.1%)	3 (5.7%)

^**^Value applies to patients with surviving grafts only

*NA*, not available; *TEA*, total elbow arthroplasty; *IPA*, interposition arthroplasty; *ORIF*, open reduction and internal fixation

## Discussion

IPA of the post-traumatic osteoarthritic elbow provides an alternative to TEA in young, high-demand patients. However, syntheses of the functional outcomes are lacking.

Our systematic review included 67 patients treated for post-traumatic osteoarthritis with either fascia lata autografts or Achilles tendon allografts. To ensure a homogeneous and thus adequately comparable study cohort, patients with primary, inflammatory, or rheumatoid osteoarthritis and revisions were excluded.

Our results indicate that IPA of the post-traumatic osteoarthritic elbow can provide satisfactory results, given graft survival. The post-operative MEPS was available in 49 patients with surviving grafts and averaged 69 points. On average, this equates to an acceptable result (“fair”). With regard to range of motion of both, flexion–extension and forearm rotation, individual data were available in 3 studies. In these, nevertheless, a considerable improvement could be achieved. However, due to an average follow-up of 61 months, these functional outcomes should be viewed with caution.

Aside from outcome scores, the occurrence of complications was evaluated: A 20.9% revision rate should be discussed. Concerning this, a study by Larson et al. showed promising results [12]. Nine patients with post-traumatic osteoarthritis underwent revision IPA with Achilles tendon allografts after failed primary IPA. After a mean follow-up of 5.6 years, the mean MEPS improved significantly from 49 points pre-operatively to 73 points post-operatively. The authors concluded that “revision interposition arthroplasty is an option for young, active patients with severe post-traumatic arthritis who require both mobility and durability of the elbow” [12].

Revision following an average of 9.9 years after primary IPA and associated implantation of a TEA similarly demonstrated satisfactory results in a study by Blaine et al. [13]. The authors displayed significant improvement in MEPS from an average of 32.1 points pre-operatively to 80.4 points post-operatively in 12 patients, 11 of whom had post-traumatic osteoarthritis. Ten of the 12 patients subjectively reported satisfactory results and described their pain as mild or none.

In a retrospective study, Celli et al. reported 19 patients aged ≤ 40 years with post-traumatic osteoarthritis treated with TEA and followed-up for at least five years [14]. The functional outcome was satisfactory with a mean MEPS of 84 points; however, seven patients (36.8%) needed a revision procedure. Due to the high complication rate, the authors conclude to prefer non-replacement methods such as IPA when possible. Accordingly, especially in young, active post-traumatic osteoarthritic patients, the finality of a prosthesis should be discussed critically.

A robust statement regarding the superiority of either graft (fascia lata autograft and Achilles tendon allograft) is not possible based on the current study record and remains pending. Regarding revisions, Cheng et al. found a rate of 33% (3/9), with patients treated solely with fascia lata autografts [7]. In the study by Nolla et al., who used autogenous fascia lata in two patients likewise, no individual data were given [11]. This 33% rate contrasts with an overall revision rate of 20.9% for all included patients.

There seems to be a consensus that IPA is contraindicated insofar as pain is not accompanied by elbow dysfunction [9]. In the post-traumatic collective considered here, however, pain is not isolated. Regarding this, Larson et al. demonstrated that isolated pain was associated with rather unpredictable outcomes, whereas nine of ten patients treated for elbow stiffness reported at least pain improvement [9]. The study by Laubscher et al. also showed significant pain improvement in patients with surviving graft [10].

With the exception of the study by Larson et al., where no individual data was given, an external fixator was applied in all patients. The effective value of an external fixator in preventing post-operative instability remains unresolved. The potential stability benefit of an external fixator faces the incidence of post-operative pin related problems of the external fixator. Based on the previously defined inclusion and exclusion criteria and limited individual data, it was not possible to systematically correlate pre-operative instability with post-operative outcome. However, pre-operative varus-valgus instability on the clinical examination seems to correlate significantly with unsatisfactory post-operative function scores [7, 9–11]. Larson et al. conclude: “we do not recommend this procedure when patients present with pre-operative instability on physical examination” [9]. Accordingly, IPA is thus able to increase range of motion while achieving satisfactory clinical outcomes, yet instabilities often fail to be resolved by IPA. This is especially true for the post-traumatic, young, and active patient cohort enrolled in this systematic review.

Limitations of this systematic review include the retrospective design of all studies included and the lack of prospective randomized controlled studies comparing treatment options. In addition, individual data were not always available, which made it impossible to draw conclusions about individual patients in some cases. In particular, pain and instability were recorded inconsistently and prevented systematic synthesis. In the future, it would be desirable to establish reporting guidelines that make individual studies more comparable with each other.

Nevertheless, this systematic review provides an updated survey and synthesis of the current literature and may shift attention to issues rather missed out until now. Interposition arthroplasty for post-traumatic osteoarthritis of the elbow provides satisfactory functional outcomes, provided graft survival. However, a 20.9% revision rate as well as a cumulative complication and subsequent operative treatment rate of 44.8%, in the setting of graft survival, should be discussed. Successful revision involving a repeat IPA or conversion to TEA is possible. Pre-operative varus-valgus instability seems to be associated with unsatisfactory postoperative elbow function. The superiority of either of the two reported graft methods (fascia lata autograft and Achilles tendon allograft) remains pending, and the role of an external fixator in preventing post-operative instability remains unresolved.

## Data Availability

Not applicable.
